# Supplementary material for: Calpains orchestrate secretion of annexin-containing microvesicles during membrane repair
Source: J Cell Biol. 2025 May 16;224(7):e202408159. doi: 10.1083/jcb.202408159 (PMC12083247; doi:10.1083/jcb.202408159)
Supplement: SourceData F1 — is the source file for Fig. 1. [file jcb_202408159_sourcedataf1.pdf]

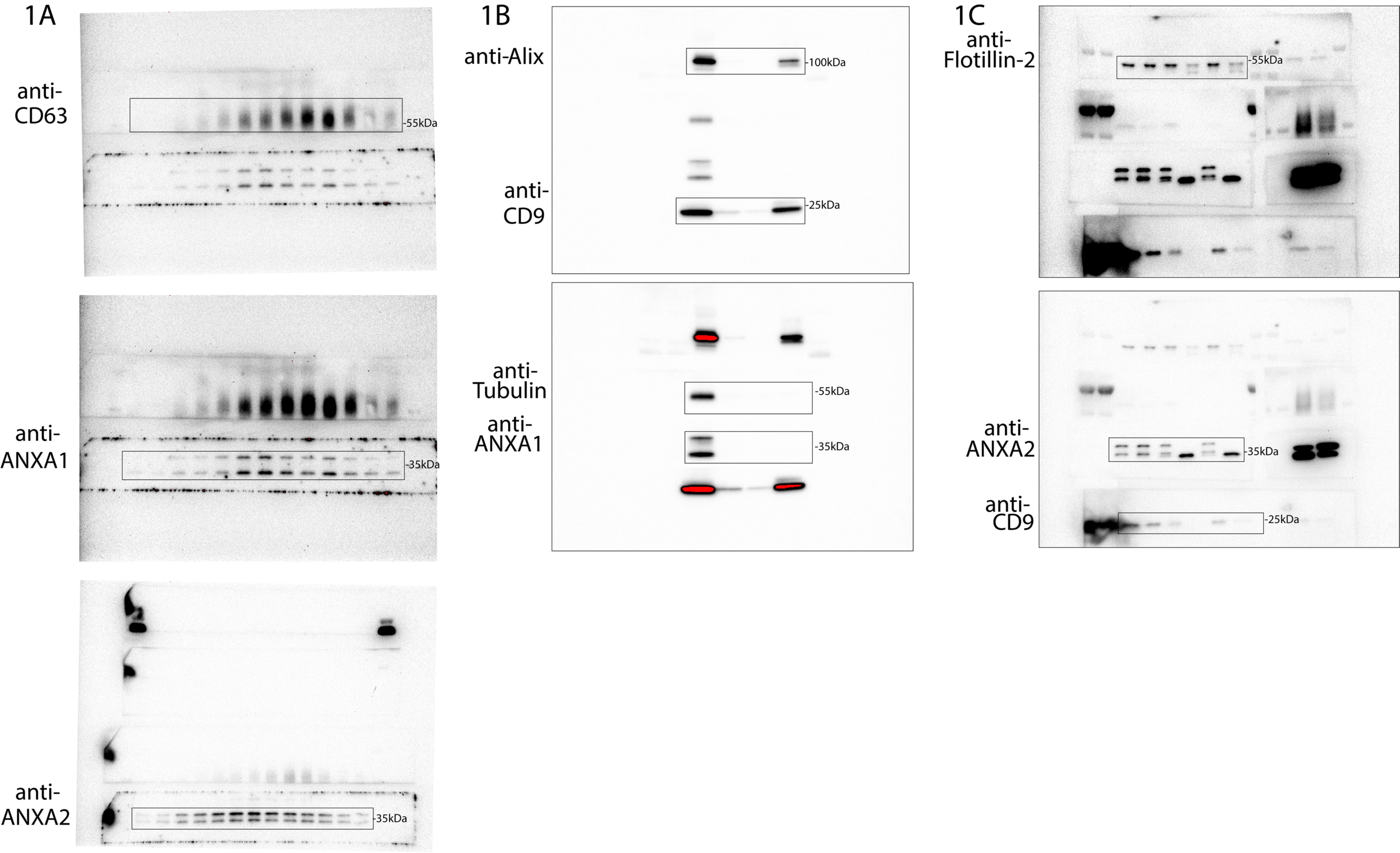
1A
anti-CD63
-55kDa
anti-ANXA1
-35kDa
anti-ANXA2
-35kDa
1B
anti-Alix
-100kDa
anti-CD9
-25kDa
anti-Tubulin
-55kDa
anti-ANXA1
-35kDa
1C
anti-Flotillin-2
-55kDa
anti-ANXA2
-35kDa
anti-CD9
-25kDa
