# Supplementary figures and images for: Calpains orchestrate secretion of annexin-containing microvesicles during membrane repair
Source: J Cell Biol. 2025 May 16;224(7):e202408159. doi: 10.1083/jcb.202408159 (PMC12083247; doi:10.1083/jcb.202408159)

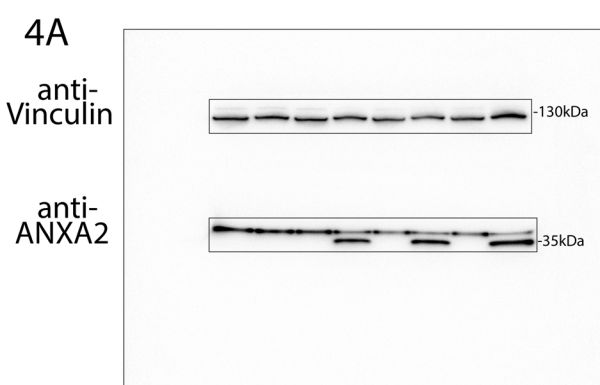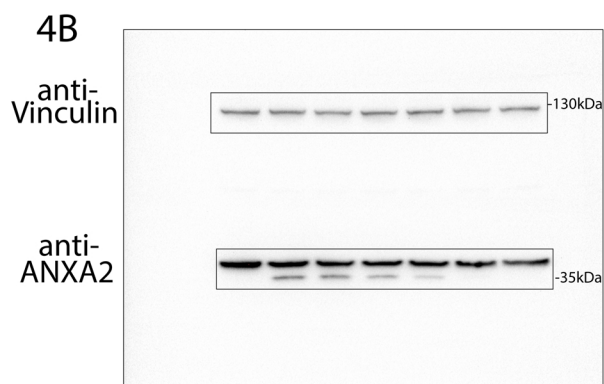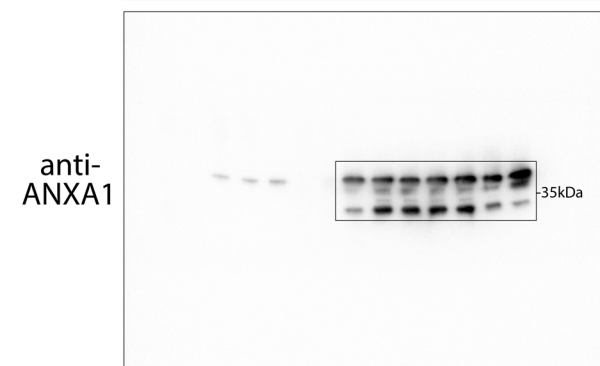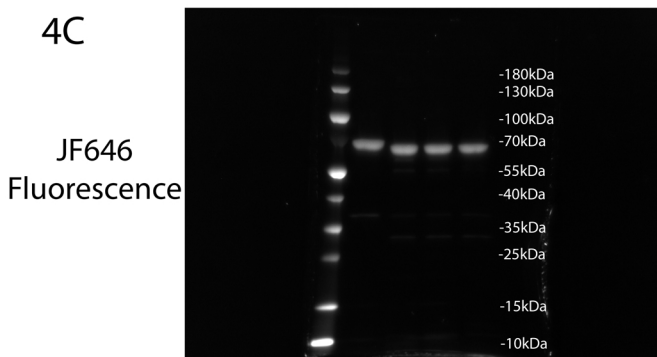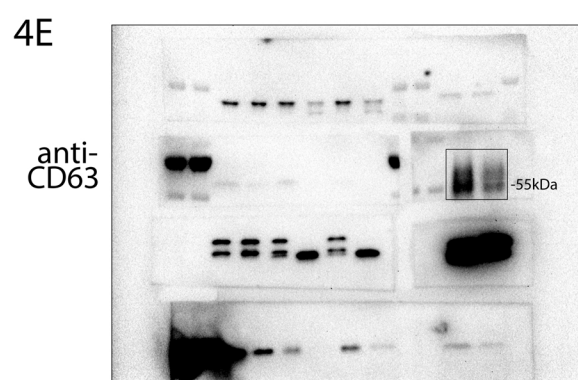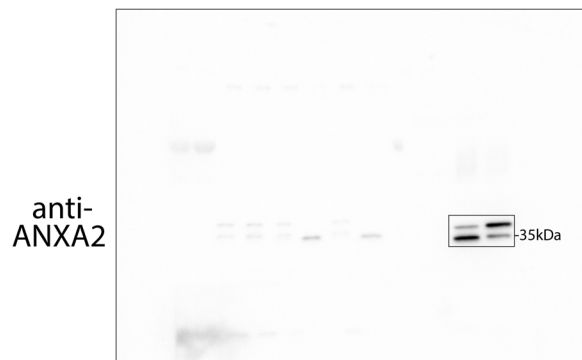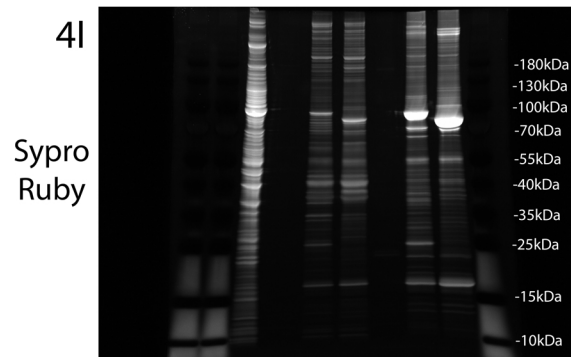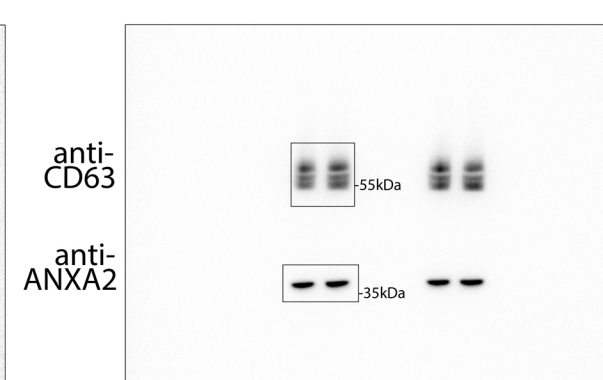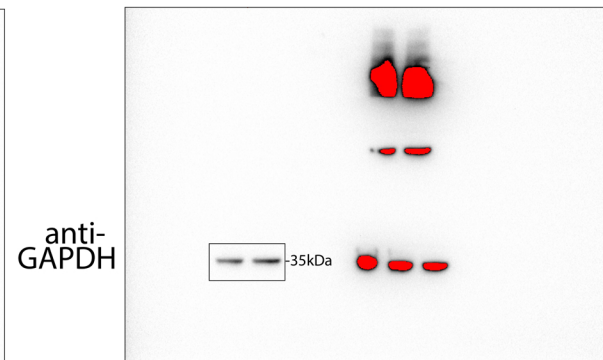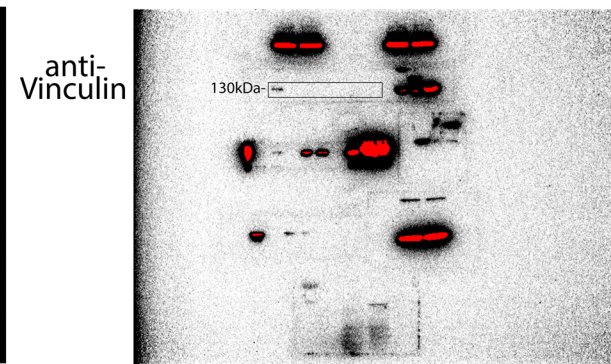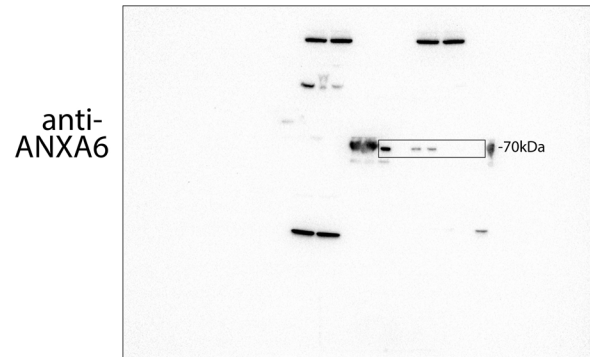

Supplement: SourceData F4 — is the source file for Fig. 4. [file jcb_202408159_sourcedataf4.pdf]

5A

anti-Vinculin

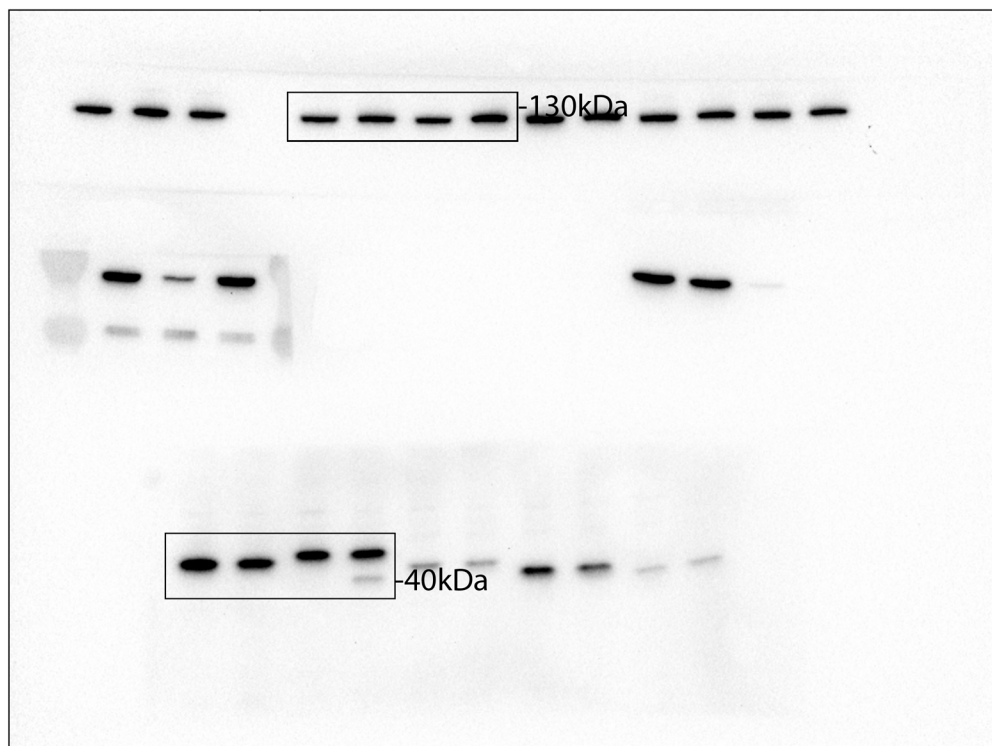

5B

anti-Vinculin

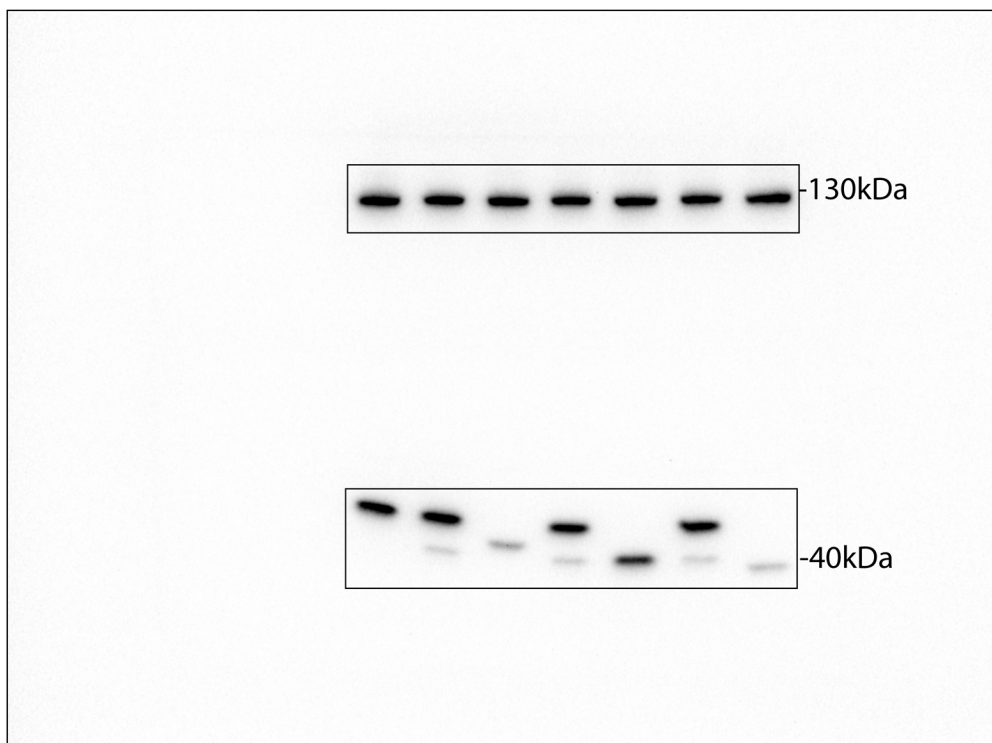

anti-HA

5D

anti-Vinculin

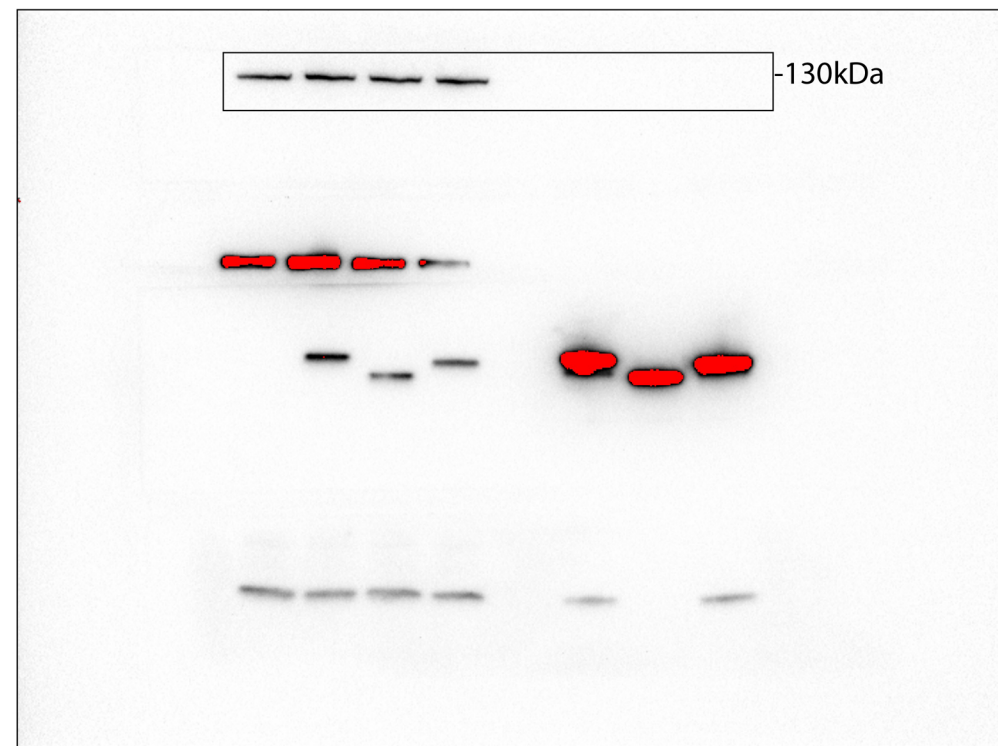

anti-HA

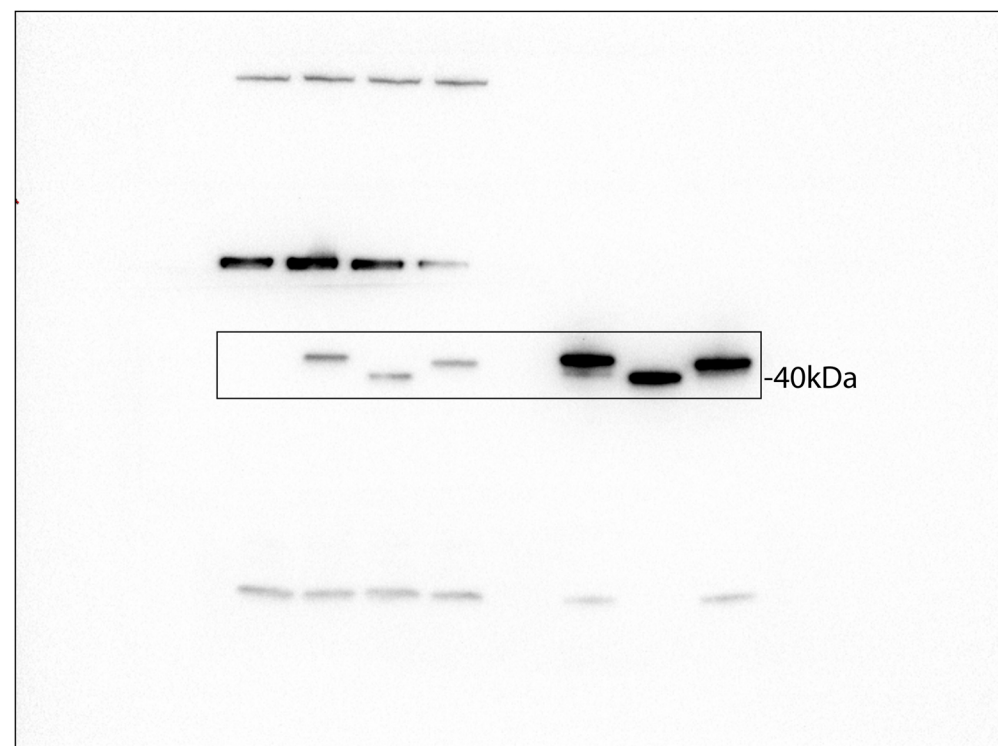

anti-S100A10

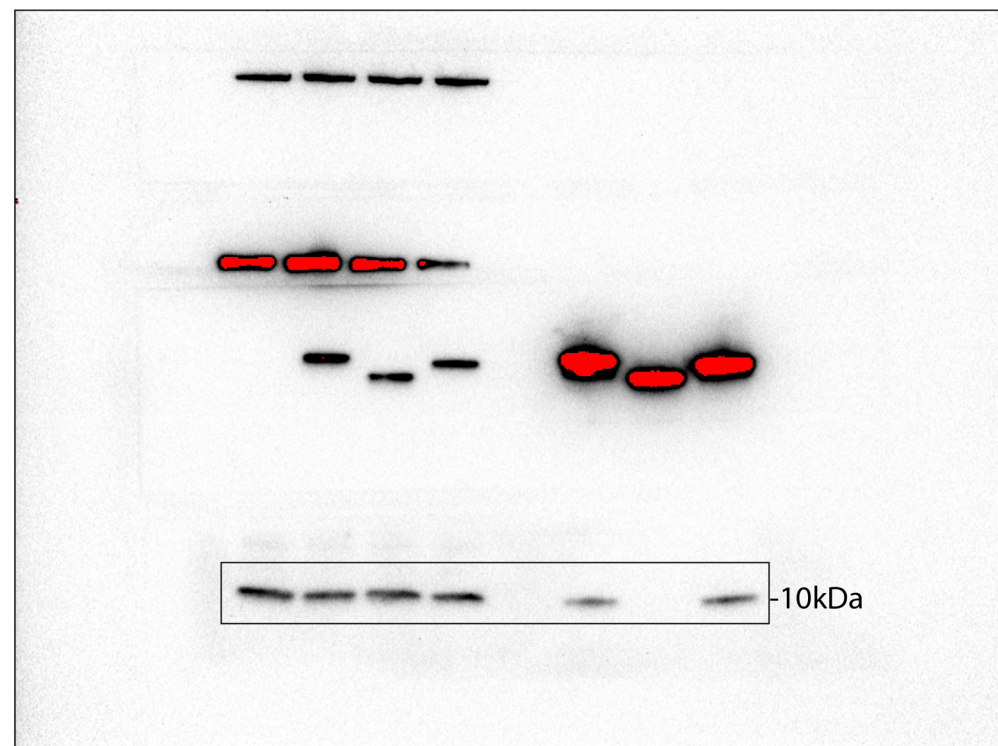

Supplement: SourceData F5 — is the source file for Fig. 5. [file jcb_202408159_sourcedataf5.pdf]

6B anti-  
Vinculin

anti-  
ANXA2

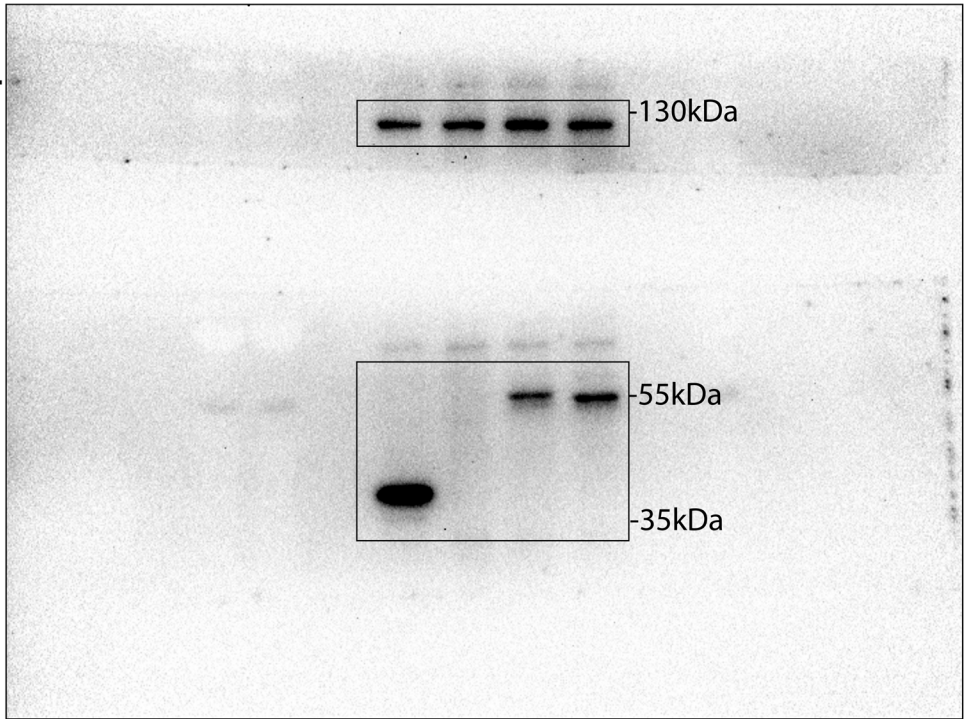

Supplement: SourceData F6 — is the source file for Fig. 6. [file jcb_202408159_sourcedataf6.pdf]

S1A

anti-Alix

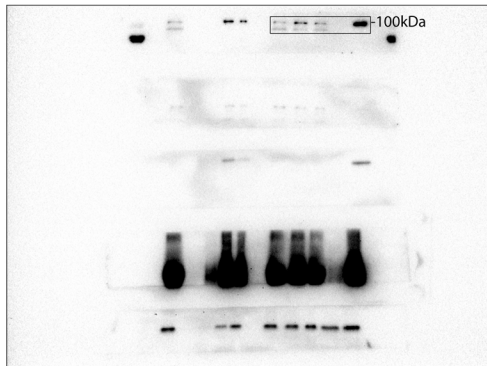

anti-  
CD63

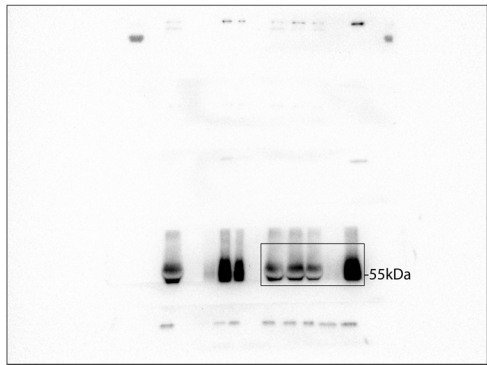

S1B

anti-  
CD63

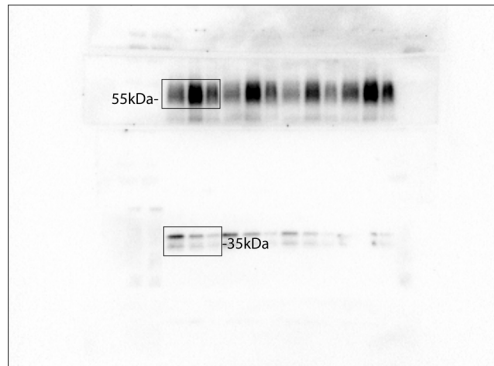

anti-  
ANXA2

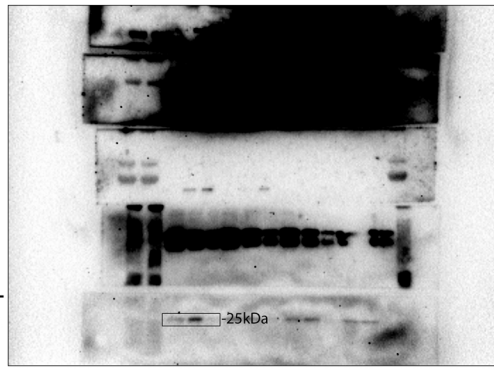

anti-  
CD9

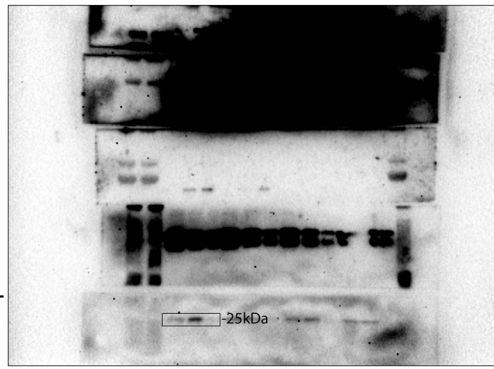

Supplement: SourceData FS1 — is the source file for Fig. S1. [file jcb_202408159_sourcedatafs1.pdf]

S2F

anti-Vinculin

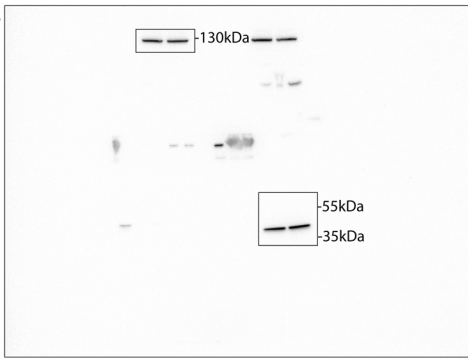

anti-ANXA2

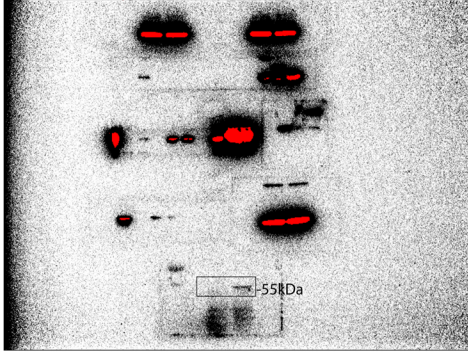

anti-Nanoluc

S2G

anti-Alix

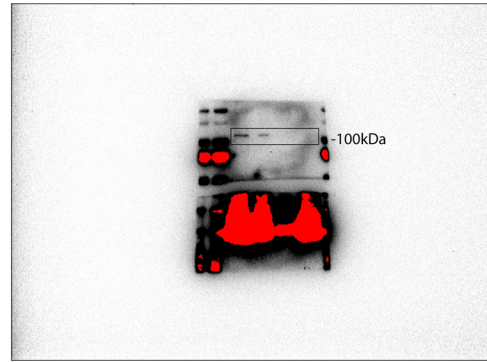

anti-CD63

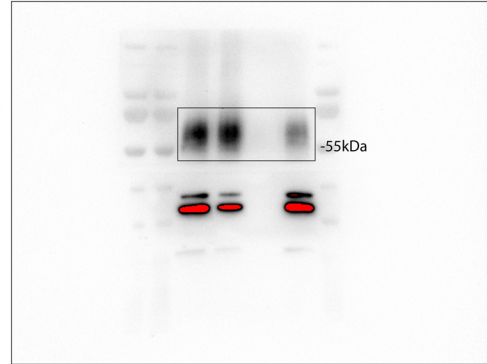

anti-ANXA1

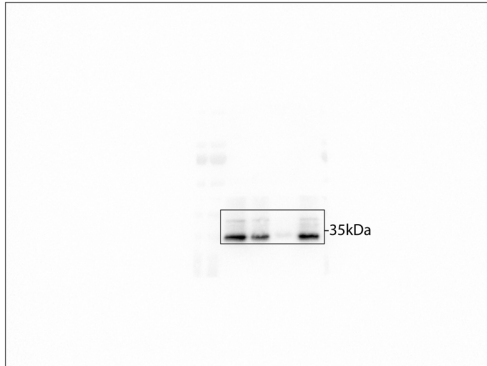

anti-ANXA2

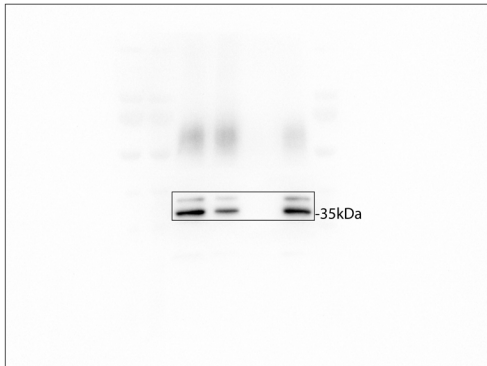

anti-CD9

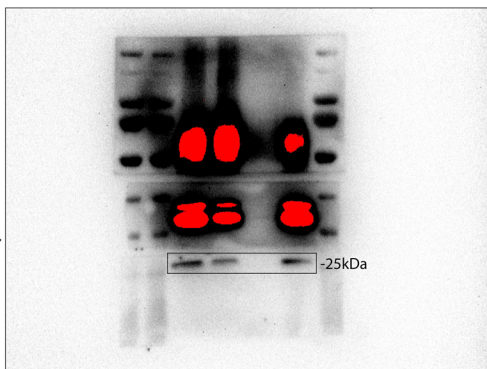

Supplement: SourceData FS2 — is the source file for Fig. S2. [file jcb_202408159_sourcedatafs2.pdf]

S3A

anti-Alix

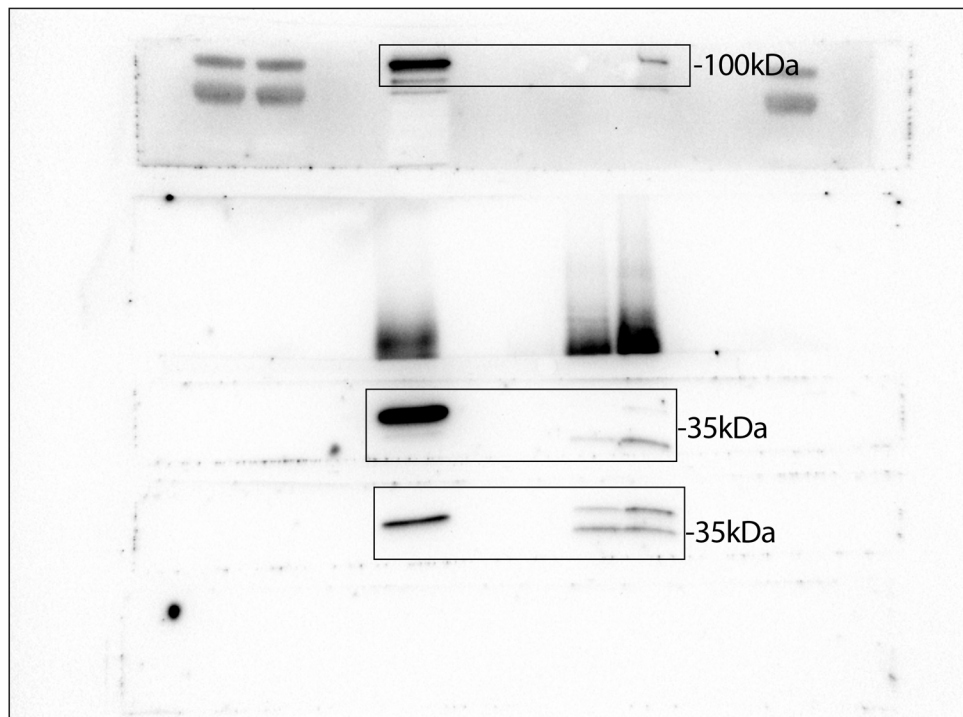

anti-  
ANXA1

anti-  
ANXA2

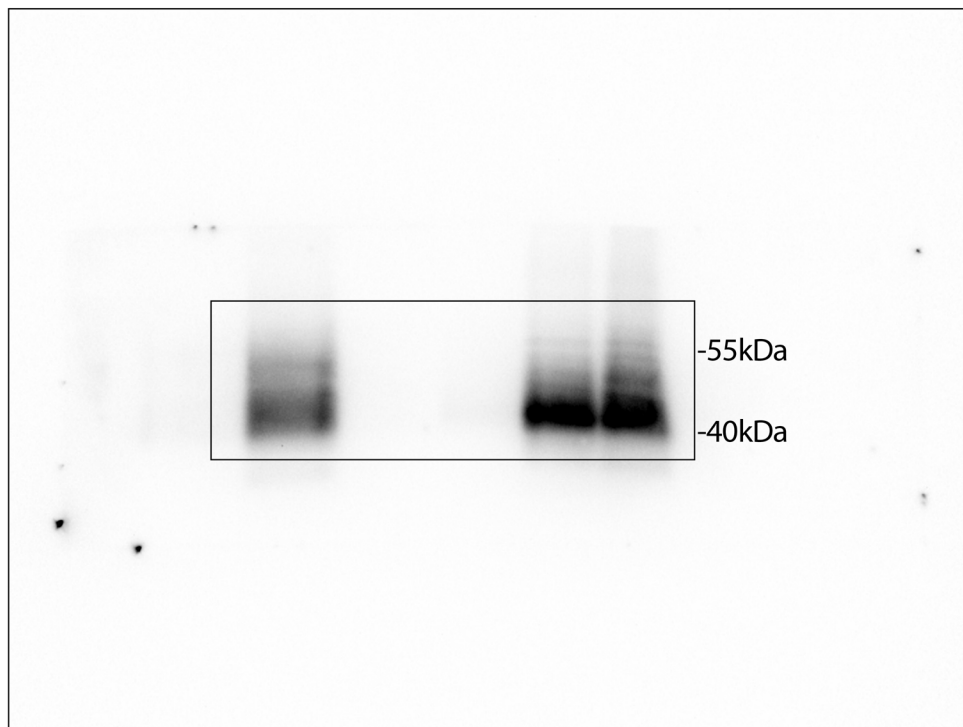

S3B

anti-  
ANXA2

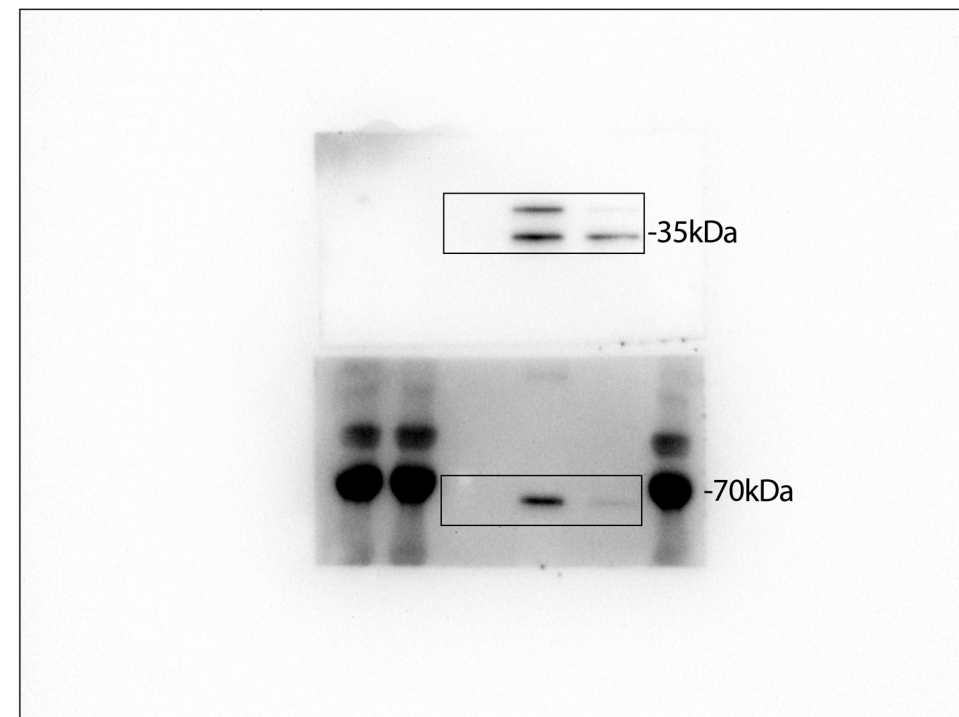

anti-SLO

Supplement: SourceData FS3 — is the source file for Fig. S3. [file jcb_202408159_sourcedatafs3.pdf]

S4A

anti-Vinculin

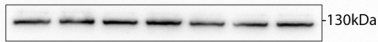

anti-ANXA2

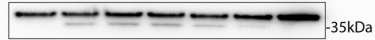

S4C

Coomassie

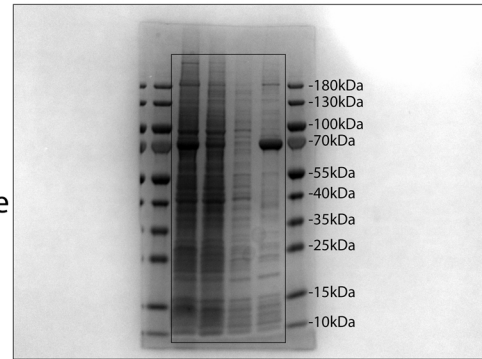

S4B

Coomassie

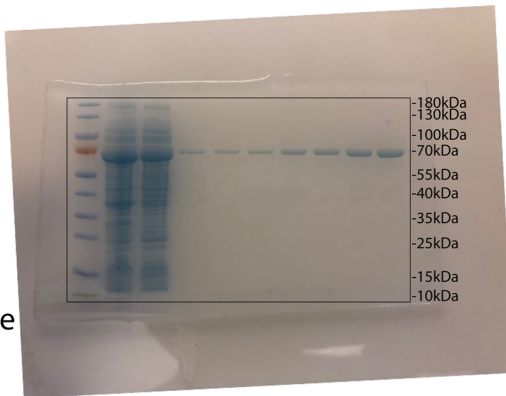

S4D

Coomassie

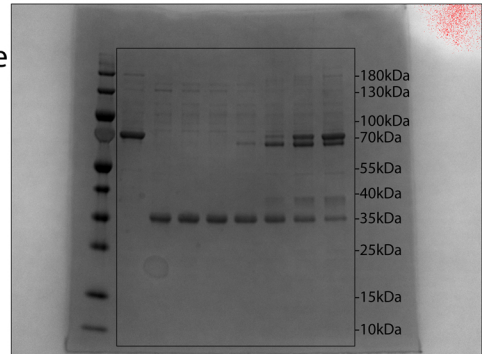

S4F

Coomassie

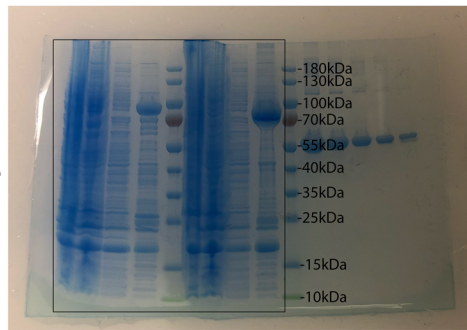

Supplement: SourceData FS4 — is the source file for Fig. S4. [file jcb_202408159_sourcedatafs4.pdf]

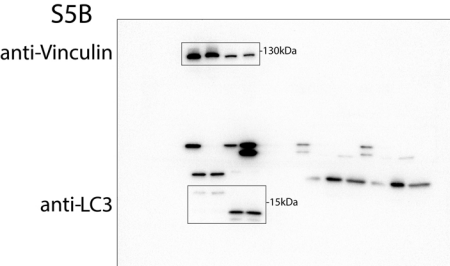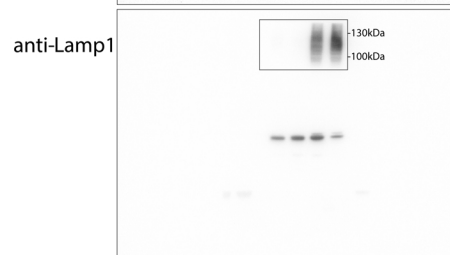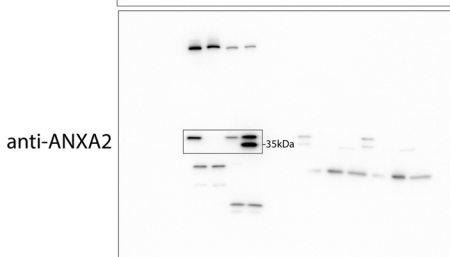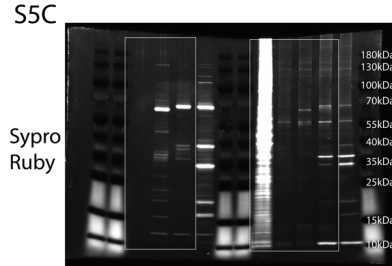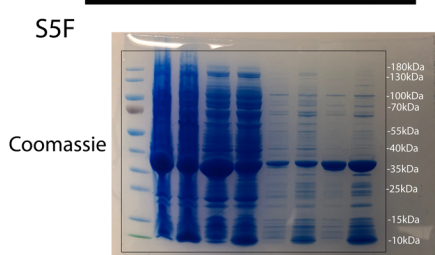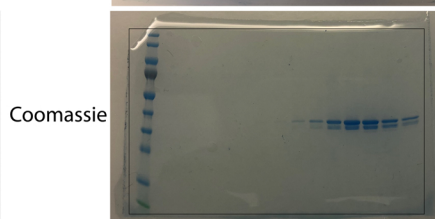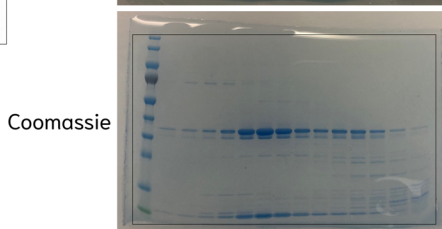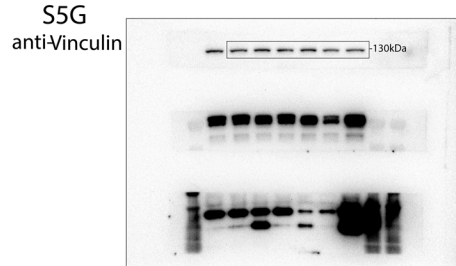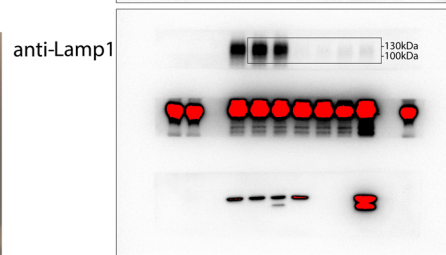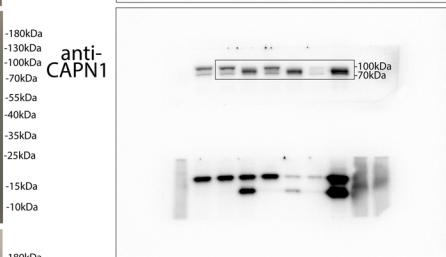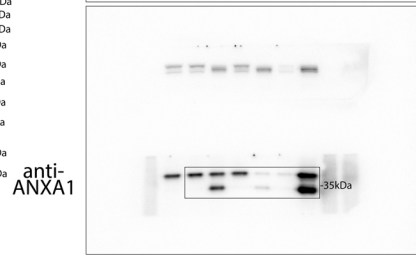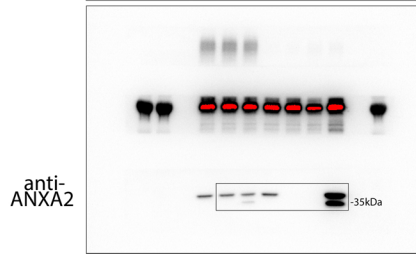

Supplement: SourceData FS5 — is the source file for Fig. S5. [file jcb_202408159_sourcedatafs5.pdf]
